# Frequency- and Phase Encoded SSVEP Using Spatiotemporal Beamforming

**DOI:** 10.1371/journal.pone.0159988

**Published:** 2016-08-03

**Authors:** Benjamin Wittevrongel, Marc M. Van Hulle

**Affiliations:** Laboratory for Neuro- and Psychophysiology, K.U. Leuven, Leuven, Belgium; Duke University, UNITED STATES

## Abstract

In brain-computer interfaces (BCIs) based on steady-state visual evoked potentials (SSVEPs) the number of selectable targets is rather limited when each target has its own stimulation frequency. One way to remedy this is by combining frequency- with phase encoding. We introduce a new multivariate spatiotemporal filter, based on Linearly Constrained Minimum Variance (LCMV) beamforming, for discriminating between frequency-phase encoded targets more accurately, even when using short signal lengths than with (extended) Canonical Correlation Analysis (CCA), which is traditionally posited for this stimulation paradigm.

## Introduction

The steady-state visual evoked potential (SSVEP) is a neurophysiological response to a periodic visual stimulus commonly gauged with electroencephalography (EEG) over the occipital cortex when using stimuli flickering at a frequency above 6 Hz but in practice lower than about 30 Hz (due to the usual 60 Hz screen refresh rate and the EEG bandwidth). The recorded EEG signals not only resonate at that frequency but also contain a number of harmonics. When considering a display with several disjoint, spatially delimited stimuli, each one flickering at a different frequency, the stimulus the subject is currently gazing at can be inferred from a spectral analysis of the recorded EEG signals. This is also the principle behind the SSVEP-based brain-computer interface (BCI) where those stimuli become selectable targets in a subject interaction paradigm. However, a simple frequency analysis technique based on the (fast) Fourier transform [1, 2] typically requires long (i.e., 3 seconds or more [3, 4]) signals to accurately discriminate targets flickering at nearby frequencies. Further studies have led to several SSVEP detection techniques that are able to work with shorter signals such as *Similarity of Background* (SOB) [5], *Minimum Energy Combination* (MEC) [6], time-domain analysis [7, 8], and the widely adopted *Canonical Correlation Analysis* (CCA) [9–12] and its variants [13, 14].

The number of frequency-coded targets is not only limited by the harmonics of the stimulus frequency, but also by the screen refresh rate: when using a 50% duty-cycle stimulation (i.e., ‘on-off’ stimulation) the usable frequencies are restricted to integer dividers of the screen refresh rate. This restriction was demoted by replacing on-off stimulation with screen luminosity modulation [15].

As an alternative to frequency coding, also phase coding of the targets has been suggested [15–21]: (a subset of) targets flicker at the same frequency but with different phase ‘lags’. However, discriminating phases is more challenging and, typically, the number of useable phases is also quite limited, especially when based on short signals [20].

Another attempt to increase the number of selectable targets is to consider for each target a unique combination of frequency and phase [22–24]. For the joint detection of frequency and phase, the CCA method was extended and shown to be useful for a high-speed BCI application [24]. We will also consider combined frequency/phase coded targets but propose a new decoding approach: spatiotemporal beamforming in combination with time-domain analysis of EEG signals. The beamformer was originally formulated as a spatial filter for radar, sonar and seismic data analysis [25]. It was also employed in EEG analysis to isolate the signal originating from a predefined brain location [26], to estimate the amplitude of an ERP component [27, 28], and to build a BCI application based on imagined movement detection [29]. Here we extend the beamformer to a spatiotemporal filter for combined frequency-phase SSVEP BCI.

## Methods

### Subjects

We recruited 21 subjects, for our experiments, 14 female and 7 male (average age 22.7 years, between 19 to 26 years). Prior to the experiment, and after being informed of its purpose and design, our subjects read and, when they agreed, signed an informed consent form previously approved by the ethical committee of our university hospital UZLeuven. All subjects had normal or corrected to normal vision and were paid for their participation. All experiments were done in a sound-attenuated, air-conditioned room.

### Interface

**Fig 1:**
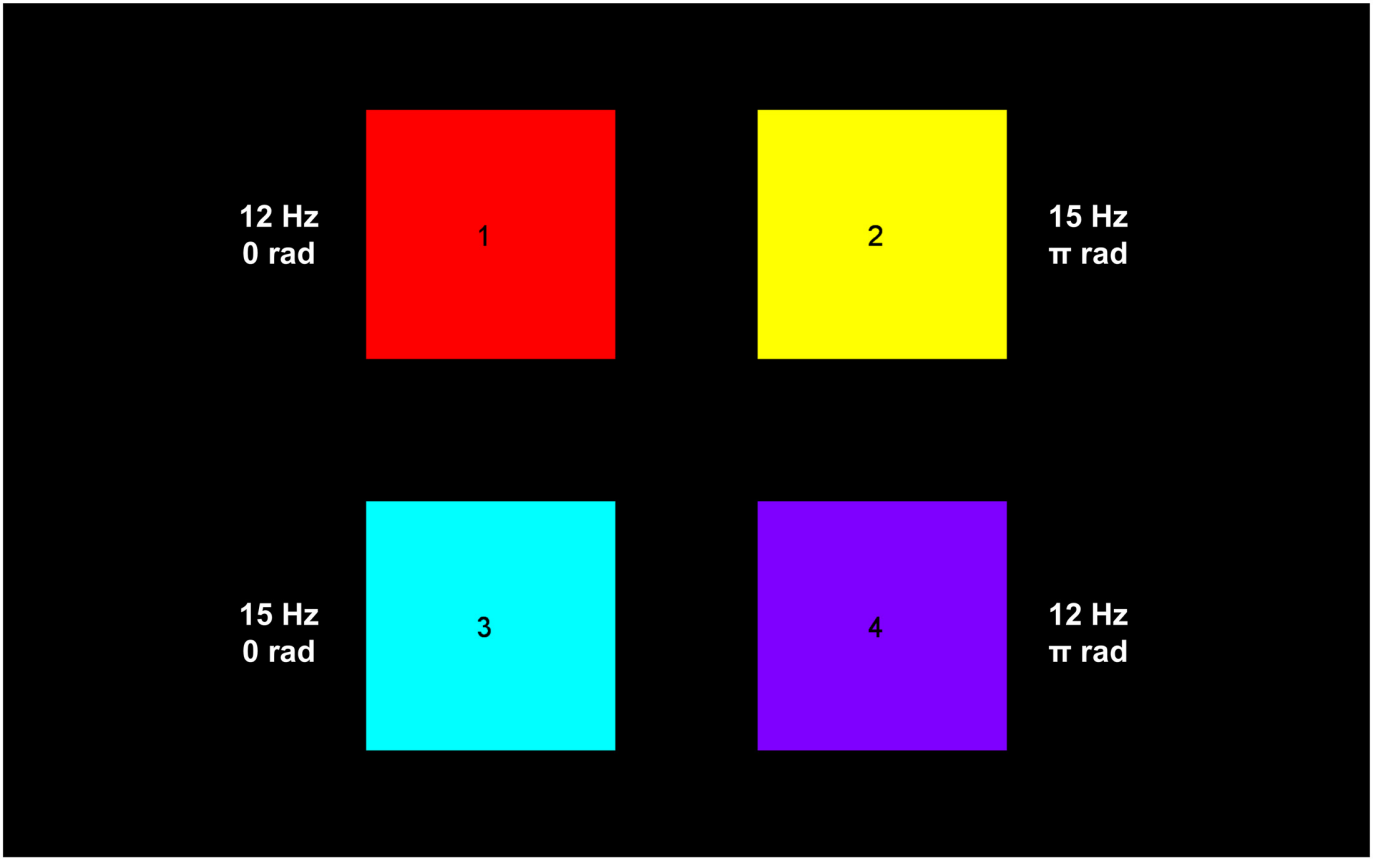
Interface used in the experiment. Each of the four squares has a unique combination of frequency and phase.

The targets consisted of four identical squares sized 9.5 × 9.5 cm, horizontally and vertically separated by 5.4 cm gap (∼4.4°) and diagonally by a 7.6 cm gap (∼6.2°), projected on an LCD computer display (sized 24.1 inch, resolution 1920×1200, 60 Hz refresh rate). Each square was assigned a different combination of frequency (12 or 15 Hz) and phase (0 or *π* radians) (Fig 1). Subjects were seated in a comfortable arm chair at a distance of approximately 70 cm from the display.

The recording session consisted of 60 trials. At the beginning of each trial, one of the squares was cued (target) with its corresponding color while the other squares were shown in gray. The subject was asked to direct his/her gaze at the cued square during the entire five-second stimulation. The trial was initiated when the subject pressed a key on the keyboard after which the all the squares regained their color. During a trial, all squares were flickering, in accordance with their frequency/phase combination, by sinusoidally modulating their luminosities using the method described in [15]. Between trials, the subject was allowed to take a break.

### Recording

EEG was recorded continuously (sampling speed 2048 Hz, common mode sense (CMS) referencing) using 32 active Ag/AgCl electrodes (BioSemi Active Two) placed according to the extended international 10-20 system. Additionally, six external electrodes were placed: two on the left and right mastoids, for further off-line re-referencing, and four around the eyes, one on the upper and lower side of the left eye (vertical), and one near the external canthus of each eye (horizontal), for electro-oculogram recording (EOG, bi-polar recording). Except for these external ones, all electrodes were mounted in the electrode cap that was placed on the subject’s head. Conductive gel was applied in each of the electrode holes, as well as on the surface of the external electrodes to reduce electrode impedance. Prior to the main experiment, an EOG calibration session was performed to offline remove eye movements and blinks, using the method described in Croft & Barry [30].

### Processing

The recordings were re-referenced offline from a CMS reference to the average of both mastoid signals and the EOG signal used to remove eye artifacts following the method of Croft and co-workers [30]. The corrected signals were bandpass filtered between 5 and 20 Hz using a 4th order Butterworth filter and then cut into five-second epochs, time-locked to the stimulation onset. Finally, the epochs were downsampled to 512 Hz, and labeled according to the color cued square. For each subject, 60 five-second epochs were extracted and stored for further analysis.

### Analysis

#### Time-domain approach

**Fig 2:**
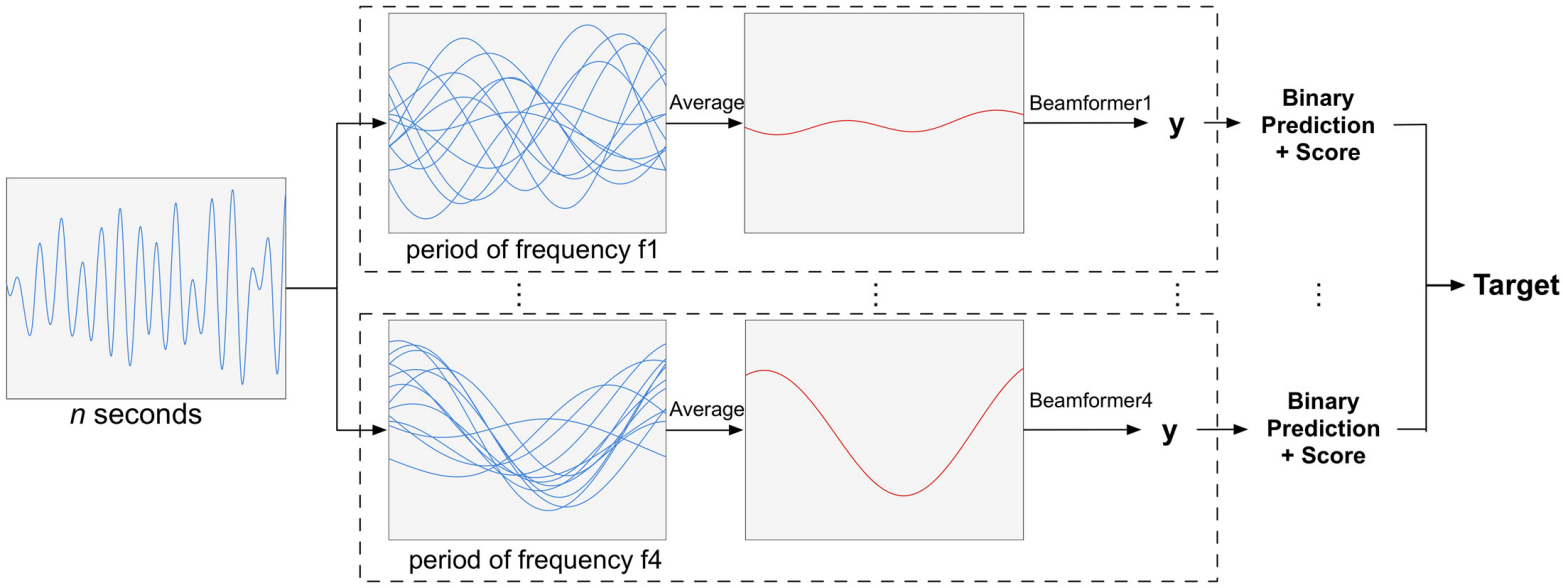
Concept behind the classification of a single epoch. Note that the actual analysis is done on a spatiotemporal epoch, involving multiple electrodes.

In order to discriminate between the frequency-and-phase encoded targets, we adopt a time-domain approach [7, 8]: each epoch is cut into consecutive, non-overlapping and identical segments with lengths equal to a single period of stimulus frequency *f* (blue traces in Fig 2). As we are using frequencies of 12 and 15 Hz, we have two such lengths, 83 and 67 ms, respectively. Then, we compute averages (red traces in Fig 2) When the stimulus frequency of the target that is gazed at matches one of the assumed stimulus periods, the corresponding average trace will be periodic, else it will be (almost) flat.

#### Beamforming

The beamformer starts from an activation pattern (i.e., a template of the signal-of-interest) that represents the EEG response to a particular frequency-phase combination (see further on how activation patterns are constructed), and transforms it into a weight vector (i.e., a multivariate filter), so as to optimally isolate the the targeted response from noise and possible non-target related activity, by taking into account the information contained in the covariance matrix estimated from the entire data. As the signals-of-interest differ among targets, each target has its own template with length equal to a single period of the target’s stimulus frequency and thus also its own beamformer. The latter is applied then to an averaged segment of the same length. As the templates differ among targets, each target has its own template and thus also beamformer that is applied to an averaged segment with length equal to a single period of the target’s stimulus frequency. The following explanation therefore applies to each target individually. Let matrix $A\in R^{m\times n}$ be the spatiotemporal activation pattern of a given frequency-phase combination, where *m* represents the number of electrodes and *n* the number of samples. Note that *n* depends on the segment length (given the sampling rate which is fixed), and thus on the target frequency (but not its phase).

While the beamformer was originally formulated as a spatial filter, we extend it to a spatiotemporal filter. We adopt the formulation of van Vliet and coworkers [27]. A Linearly Constrained Minimum Variance (LCMV) spatial beamformer $w_{sp}\in R^{m\times 1}$ minimizes the variance of the beamformer output $w_{sp}^{⊺}S$:

$$\underset{w_{sp}}{arg min}w_{sp}^{⊺}S{\left(w_{sp}^{⊺}S\right)}^{⊺}\Rightarrow \underset{w_{sp}}{arg min}w_{sp}^{⊺}\Sigma_{sp}w_{sp},$$ (1)

where $\Sigma_{sp}\in R^{m\times m}$ is the spatial covariance matrix of the EEG segment $S\in R^{m\times n}$. By adding the linear constraint:

$$a_{sp}^{⊺}w_{sp}=1,$$ (2)

where $a_{sp}\in R^{m\times 1}$ is the spatial activation pattern, we avoid trivial solutions of Eq (1), and signals that are similar to **a**_*sp*_ will be mapped to a value close to 1, allowing for an easy measure of similarity. The solution of Eq (1) under constraint Eq (2) can be found using the method of Lagrange multipliers [26]:

$$w_{sp}=\frac{\Sigma_{sp}^{-1}a_{sp}}{a_{sp}^{⊺}\Sigma_{sp}^{-1}a_{sp}}.$$ (3)

The spatial beamformer can be expanded to a spatiotemporal variant as follows. Let *r* be the number of segments, $X\in R^{r\times (mn)}$ a matrix where each row is obtained by concatenating the rows of a corresponding segment **S**_*i*_ (*i* = 1..*r*), $\Sigma \in R^{\left(mn)\times (mn\right)}$ the covariance matrix of $X^{⊺}$, and $a^{⊺}\in R^{1\times (mn)}$ a vector containing the concatenated rows of **A**. The spatiotemporal LCMV beamformer $w\in R^{(mn)\times 1}$ with the linear constraint $a^{⊺}w=1$ can now be calculated as:

$$w=\frac{\Sigma^{-1}a}{a^{⊺}\Sigma^{-1}a},$$ (4)

and applied to the data as a simple weighted sum:

y=sw, (5)

where $s\in R^{1\times (mn)}$ indicates the concatenated rows of a segment **S**.

Another way to obtain a spatiotemporal filter is to apply a spatial beamformer $w_{sp}\in R^{m\times 1}$ followed by a temporal beamformer $w_{t}\in R^{n\times 1}$. Let $B\in R^{r\times n}$ be the matrix containing the results of the spatial filter **w**_*sp*_ applied to the EEG segments **S**_*i*_:

$$B=\left(\begin{array}{c} w_{sp}^{⊺}S_{1}\\ w_{sp}^{⊺}S_{2}\\ \vdots \\ w_{sp}^{⊺}S_{r} \end{array}\right).$$ (6)

$\Sigma_{t}\in R^{n\times n}$, the covariance matrix of $B^{⊺}$, together with a temporal activation pattern $a_{t}\in R^{n\times 1}$, allows for a temporal beamformer to be obtained as follows:

$$w_{t}=\frac{\Sigma_{t}^{-1}a_{t}}{a_{t}^{⊺}\Sigma_{t}^{-1}a_{t}}.$$ (7)

By consecutively applying the beamformers constructed in Eqs (3) and (7) to the segment under consideration, a spatiotemporal beamformer is obtained:

$$y=w_{sp}^{⊺}Sw_{t}.$$ (8)

We further refer to Eq (5) as the spatiotemporal beamformer (stBF) and to Eq (8) as the chained beamformer (chBF).

#### Activation Patterns

Since each frequency-phase combination is expected to evoke a different EEG response, activation patterns need to be calculated for each target *i* (∈[1..4]). Let $E_{i}\in R^{m\times n\times r}$ be the target epochs (i.e., the epochs during which the subject was focussing on target *i*). Each epoch in $E_{i}$ is then cut into segments, using the time-domain approach described above, where frequency *f* is the stimulation frequency of target *i*. The spatiotemporal activation pattern **A**_*st*[*i*]_ is then calculated by averaging the obtained segments.

As spatial activation pattern for target *i*, the column of **A**_*st*[*i*]_ for which channel *Oz* reaches its maximal value is selected:

$$a_{sp[i]}=A_{st[i]}\left[*,t\right]$$ (9)

where

$$t=\underset{t}{arg max}A_{st[i]}\left[Oz,t\right].$$ (10)

Channel *Oz* was chosen since it can be expected that the SSVEP response is strongest at this channel.

To calculate the temporal activation pattern **a**_*t*[*i*]_ for square *i*, all epochs in $E_{i}$ are spatially filtered using a beamformer that was constructed with **a**_*sp*[*i*]_ as activation pattern. The covariance matrix used for constructing this beamformer is also estimated on $E_{i}$. The spatially filtered target epochs are then cut into segments using the time-domain approach where the frequency *f* is set to the stimulation frequency of target *i*. The temporal activation pattern is given by the average of these segments. The pseudo-code of this algorithm is shown in Algorithm 1.

**Algorithm 1:** Calculate Activation Patterns

1: **for** *i* ≔ 1 .. 4 **do**

2:  *f*_*i*_← stimulation frequency of target *i*

3:  $E_{i}\leftarrow$ target epochs for target *i*

4:  Cut epochs in $E_{i}$ into segments, using *f*_*i*_

5:  **A**_*st*[*i*]_← average segments

6:  **a**_*sp*[*i*]_← select column of **A**_*st*[*i*]_, using Eqs (9) and (10)

7:  Build beamformer with **a**_*sp*[*i*]_ and $E_{i}$

8:  Filter all epochs in $E_{i}$ with beamformer

9:  Cut filtered epochs into segments, using *f*_*i*_

10:  **a**_*t*[*i*]_← average segments

11: **end for**

#### Classifiers

Training the beamformer-based classifier involves the construction of four beamformers and ‘training’ four binary (one-vs-all) sub-classifiers, one for each target. The activation patterns and the beamformers were constructed as described earlier in this section. The feature space of the sub-classifiers is given by the output of the beamformers and is therefore one-dimensional. A threshold is applied to this output in order to discriminate between the target-(positive class) and the non-target (negative class) epochs (binary classification). Using a stratified four-fold cross-validation (i.e., in each fold, all labels occur equally frequent) for the training data, we determined for each sub-classifier the optimal threshold by using a Receiver Operating Characteristic (ROC) analysis. (An ROC curve shows the binary classification performance when varying the threshold.) Since the maximum classification performance could be reached for multiple thresholds (equal ROC points or points on the maximal iso-performance line), we took the median of those thresholds as final threshold.

Classification of an epoch is performed by independently applying each sub-classifier (and its necessary preprocessing, i.e., cutting into segments) to obtain four binary predictions and scores. (Fig 2) The binary prediction is based on the threshold of the respective sub-classifier and the score is equal to the *y*-value after applying the respective beamformer. From the sub-classifiers that return a positive prediction, the one having the highest score is taken as winner. In case of no positive predictions, the winner is determined by the sub-classifier returning the highest score.

To estimate the performance of the beamformer-based classifier, we apply a stratified five-fold cross-validation scheme. Because of the relatively high inter-subject variability of EEG responses to SSVEP stimulation [31], the analysis is run for each subject separately.

We compare our beamformer to an extension of the popular Canonical Correlation Analysis (CCA) method [24], with the number of harmonics set to three. To the best of our knowledge, when using short time windows in a frequency-phase SSVEP setting, the most reliable classification performance has been reported for the extended CCA method.

#### Parameter sensitivity

We assess the performance of the classifiers with different epoch lengths, from 250 ms to 3 s in steps of 250 ms. For each epoch length *l*, we select a window from 0.120 to 0.120+*l* seconds from the original five-second epochs, and run the analysis with these reduced epochs. With longer epoch lengths, more segments can be extracted, leading to more reliable and easier to discriminate averages (see also the discussion section). The first 100-150 ms of each trial corresponds to the latency of the brain to SSVEP stimulation. [32, 33] Including this in our analysis might lower performance, hence, we chose to start from 120 ms post stimulus-onset. Note that this was also used in the Nakanishi study we consider in our comparison [24]

Since SSVEP is a visual stimulation paradigm, it can be expected that primarily electrodes over the occipital pole are relevant. However, including channels surrounding the occipital cortex might provide information about noise sources and thus lead to a more accurate filter [34]. On the other hand, a smaller number of channels reduces the dimensionality of the spatiotemporal filter, which is expected to increase performance (less parameters to be estimated, cf., curse of dimensionality [35, 36]). To test the influence of the number of channels, we additionally run the analysis using a reduced electrode setup, one with 10 channels (Ch_env_ = {*Fz, Cz, Pz, P3, P4, PO3, PO4, O1, Oz* and *O2*}) and one with only three occipital channels (Ch_occ_ = {*O1, Oz* and *O2*}). The complete 32-channel setup will be referred to as Ch_full_.

In addition to the sampling rate of 512 Hz, we also ran the analysis using further downsampled epochs, and assessed their influence on the prediction performance. Lower sampling rates lead to less samples per segment and, thus, also to a lower dimensionality of the covariance matrices used when constructing the beamformers. The downsampling rates tested are 256 and 128 Hz, and the number of samples per segment are summarized in Table 1.

**Table 1:** Segment lengths (in samples) for different frequencies. T corresponds to one period of a given frequency. Segments are input to the beamformers.

Sample Rate (Hz)	T[12Hz] (samples)	T[15Hz] (samples)
512	42	34
256	21	17
128	10	8

#### Statistics

Significance levels are calculated using a two-sided Wilcoxon Rank Sum Test. P-values below 0.05 are considered as significant.

## Results

### Activation Patterns

**Fig 3:**
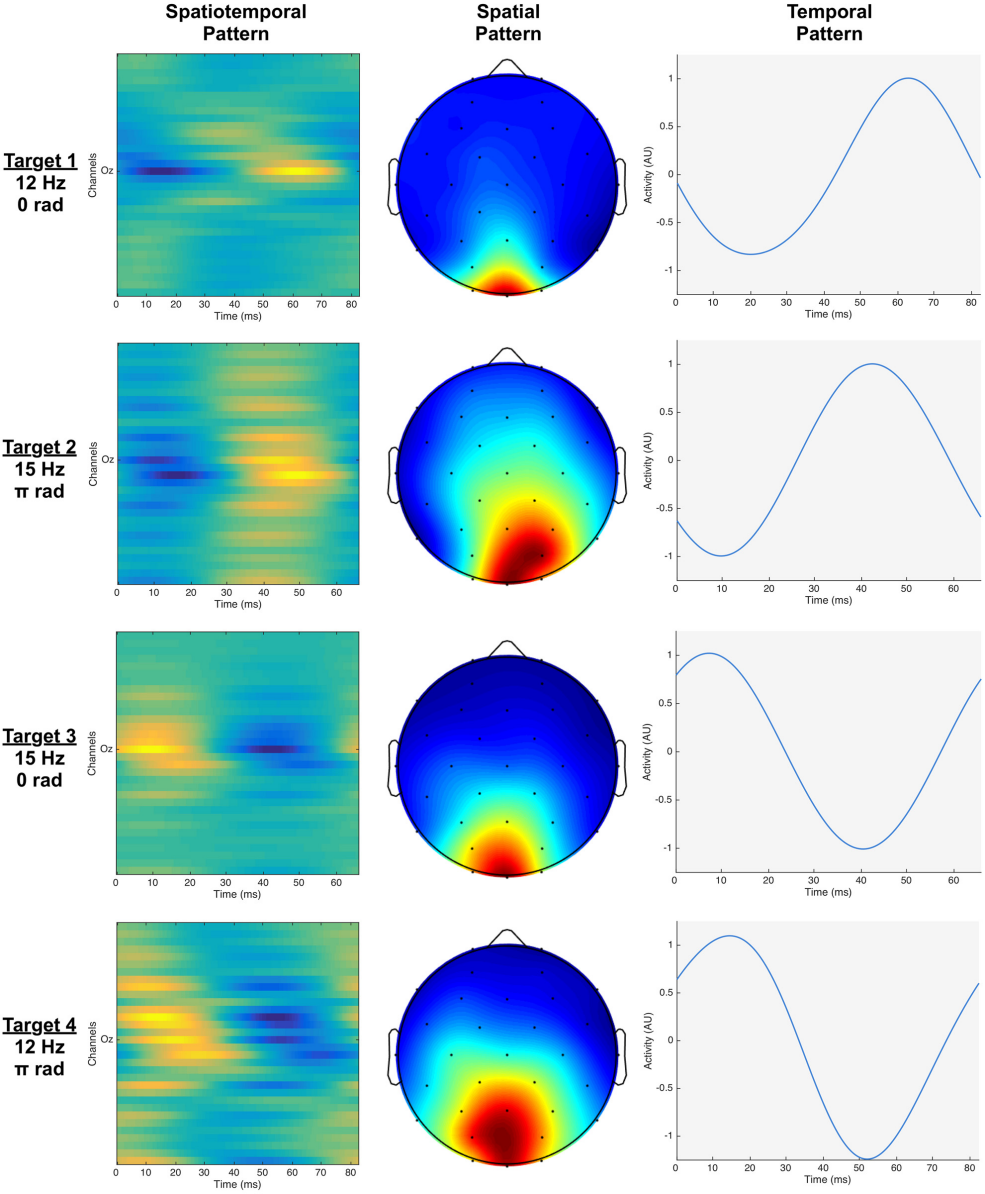
Activation patterns for subject 6. Each row corresponds to a different target. The left column shows the spatiotemporal activation patterns **A**_*st*[*i*]_ that are used for building spatiotemporal beamformers. The middle and right columns show the spatial (**a**_*sp*[*i*]_) and temporal activation patterns (**a**_*t*[*i*]_) that are employed to build the chained beamformer.

The activation patterns for one subject are shown in Fig 3. The spatial activation patterns confirm that occipital electrodes are most informative, and that parietal and parietal-occipital electrodes also can contribute to the SSVEP detection.

**Fig 4:**
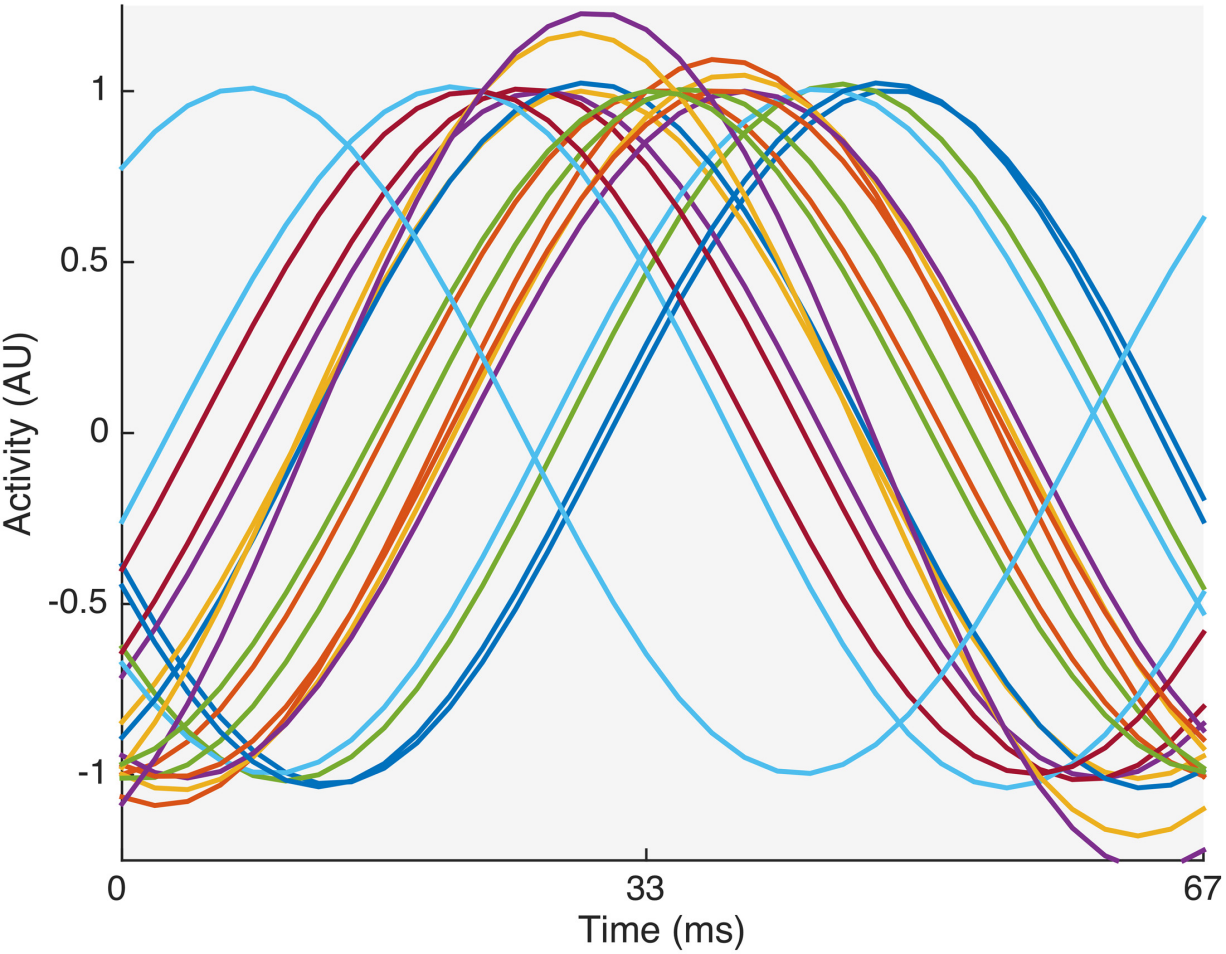
Temporal activation patterns for all subjects for target 2. Each trace corresponds to the temporal activation pattern of one subject.

When comparing the temporal patterns of all subjects for a given target (Fig 4), it is clear that they are very subject dependent. Hence, activation patterns should be determined for each subject individually rather than for a population if one is not willing to sacrifice classification performance. Therefore, we will build subject-specific beamformers and also assess their classification performance in this way.

### Classification

**Fig 5:**
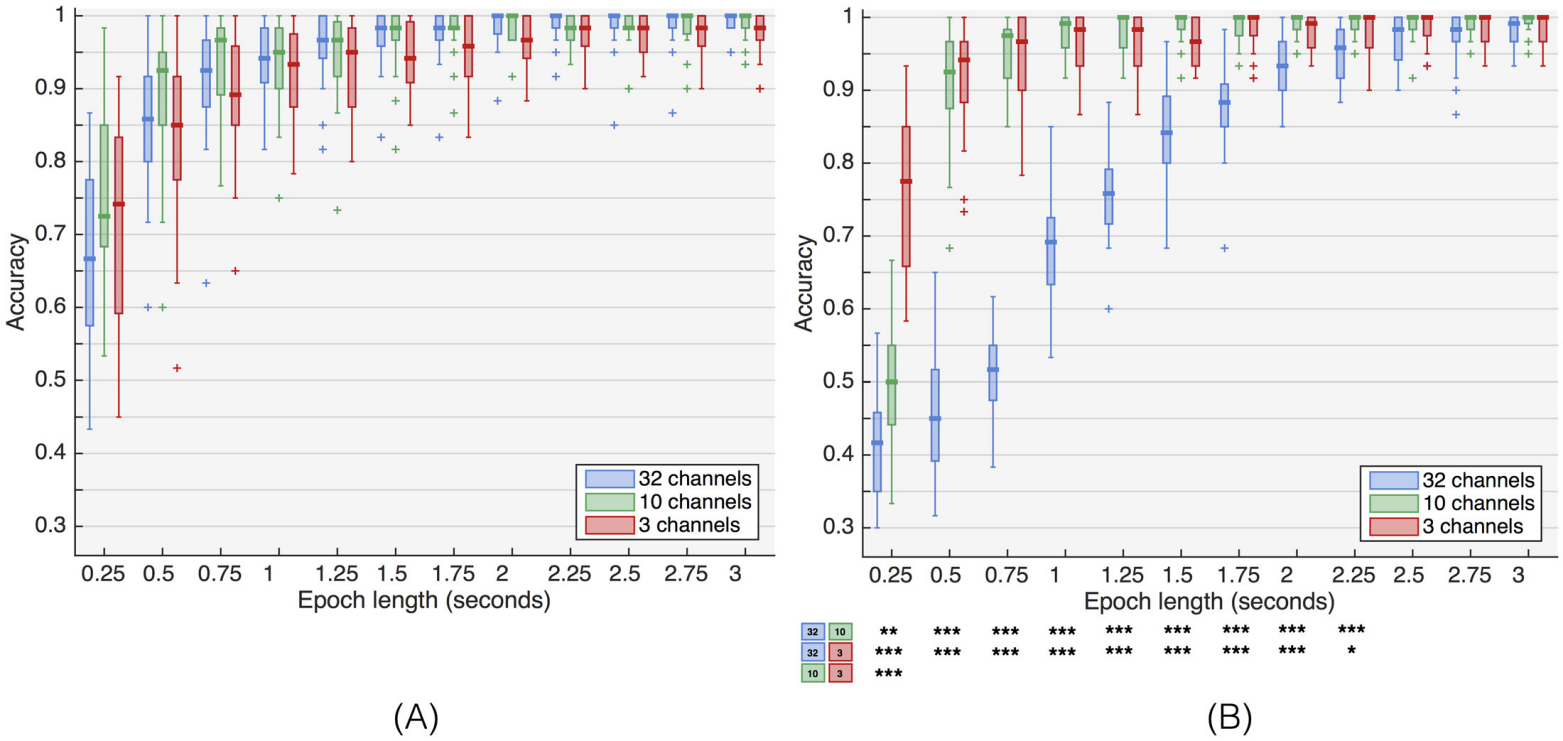
Influence of channel set on beamformer performance. (A) chained beamformer, (B) spatiotemporal beamformer. Stars indicate significance levels based on Wilcoxon Rank Sum Test: *(*p* < 0.05), **(*p* < 0.01), ***(*p* < 0.001).

Fig 5 shows the performance of the beamformers for the three channel sets, and S1 and S2 Tables summarize the statistical significance. The chained beamformer performs best using Ch_env_ on short epoch lengths (max. 1 s). Even though the differences are not statistically significant, Ch_occ_ has a lower median performance for almost all trial lengths. With stBF, both reduced channel sets significantly outperform the full channel set for epochs shorter than 2.25 seconds. Ch_env_ reaches a median accuracy of 100% for 1.25 s epoch lengths, while Ch_occ_ needs an additional 0.5 seconds to achieve similar (median) performance. Only with a very small epoch length (250 ms), Ch_env_ performs significantly worse than Ch_occ_.

**Fig 6:**
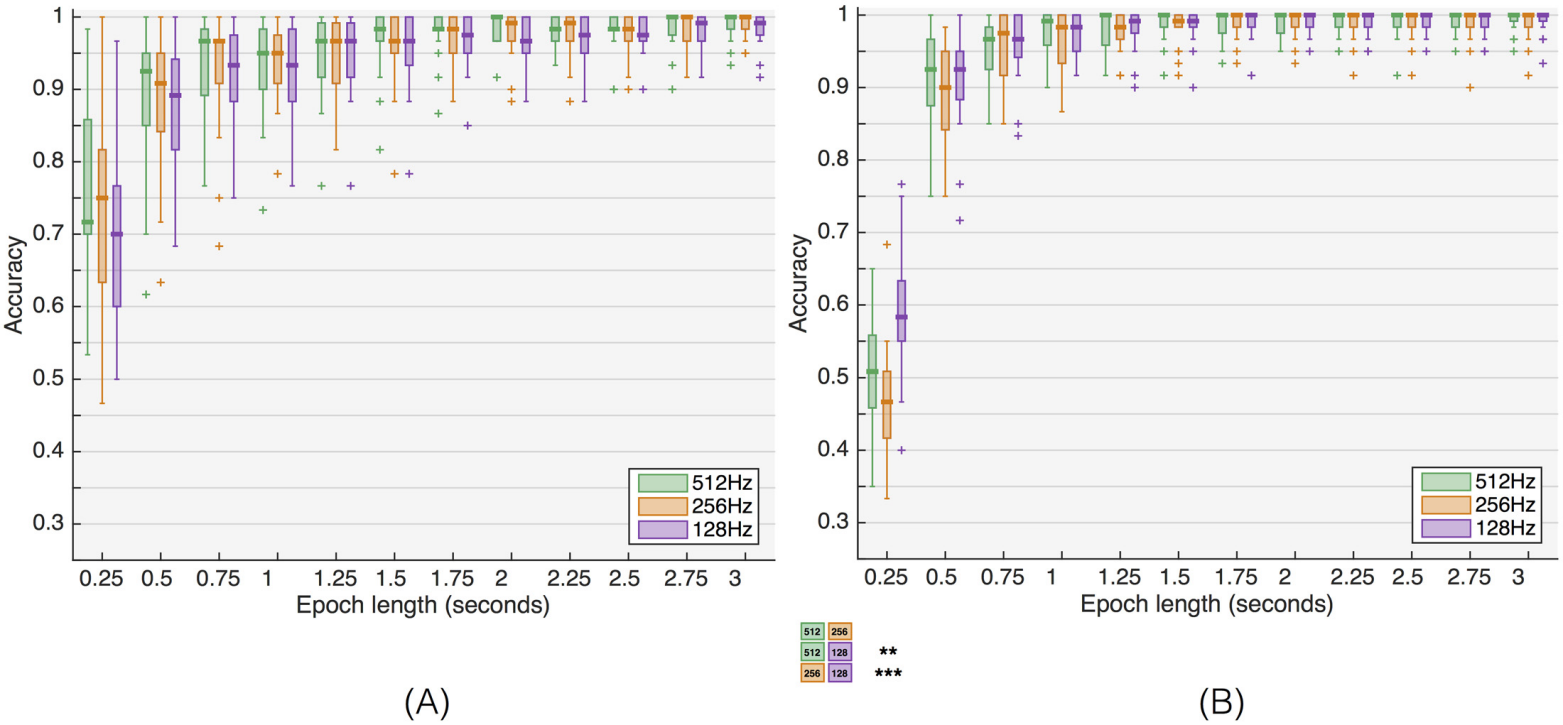
Influence of downsampling on beamformer-based classification performance. (A) chained beamformer, (B) spatiotemporal beamformer, both for channel set Ch_env_.

For Ch_env_, the effect of downsampling is shown in Fig 6 and S3 and S4 Tables. For both beamformers, there are only minor effects. None of the differences with the chBF are significant. The only significant differences with stBF are for an epoch length of 250 ms. However, in that case the median performance does not reach 70%, which is often taken as the minimum for reliable communication [37–40].

**Fig 7:**
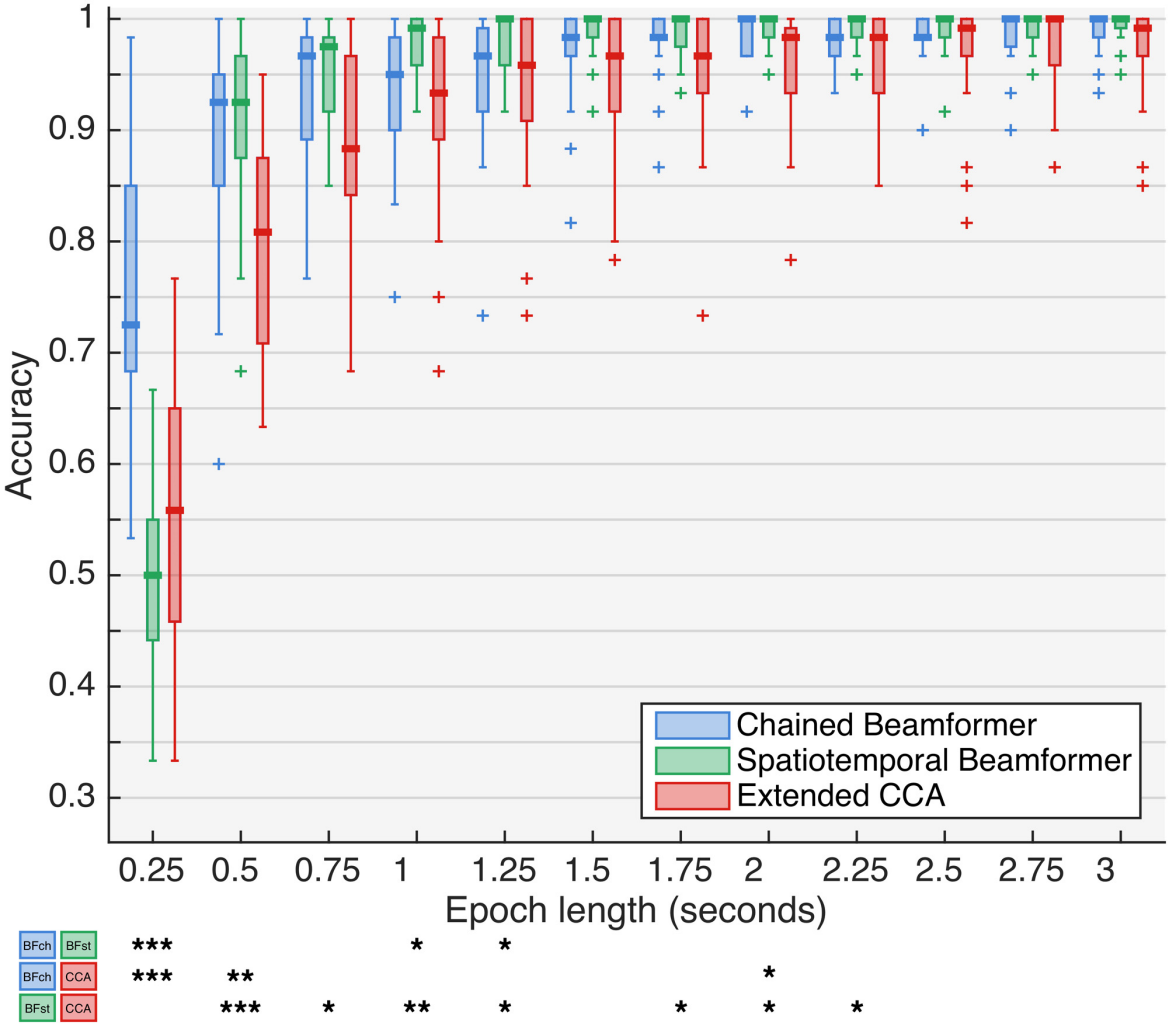
Performance of different types of classifiers. Channel set Ch_env_ and no downsampling was used. Stars indicate significance levels, based on a Wilcoxon Rank Sum Test: *(*p* < 0.05), **(*p* < 0.01), ***(*p* < 0.001).

To compare the beamformers with the extended CCA method, a sampling rate of 512Hz and Ch_env_ was used. Fig 7 shows the prediction performances, and the statistical significance is given in Classification. Except for the shortest epoch length, our beamformer-based classifiers outperform the CCA alternative. The stBF-variant is significantly better than the CCA-alternative for short epoch lengths (≤1.25 s). It is also clear that the variance of both beamformers is smaller than that of the CCA alternative. Note that the performance of the extended CCA method on our data is comparable to the original study.

The accuracies achieved by the four sub-classifiers during the analysis of the performance reported in Fig 7 are shown in S1 and S2 Figs, for the stBF- and chBF-based classifier respectively. For all targets, the sub-classifier accuracies are approximately equal. However, target 4 (i.e., 15Hz and *π* rad) has consistently the lowest detection accuracy for both beamformers.

**Fig 8:**
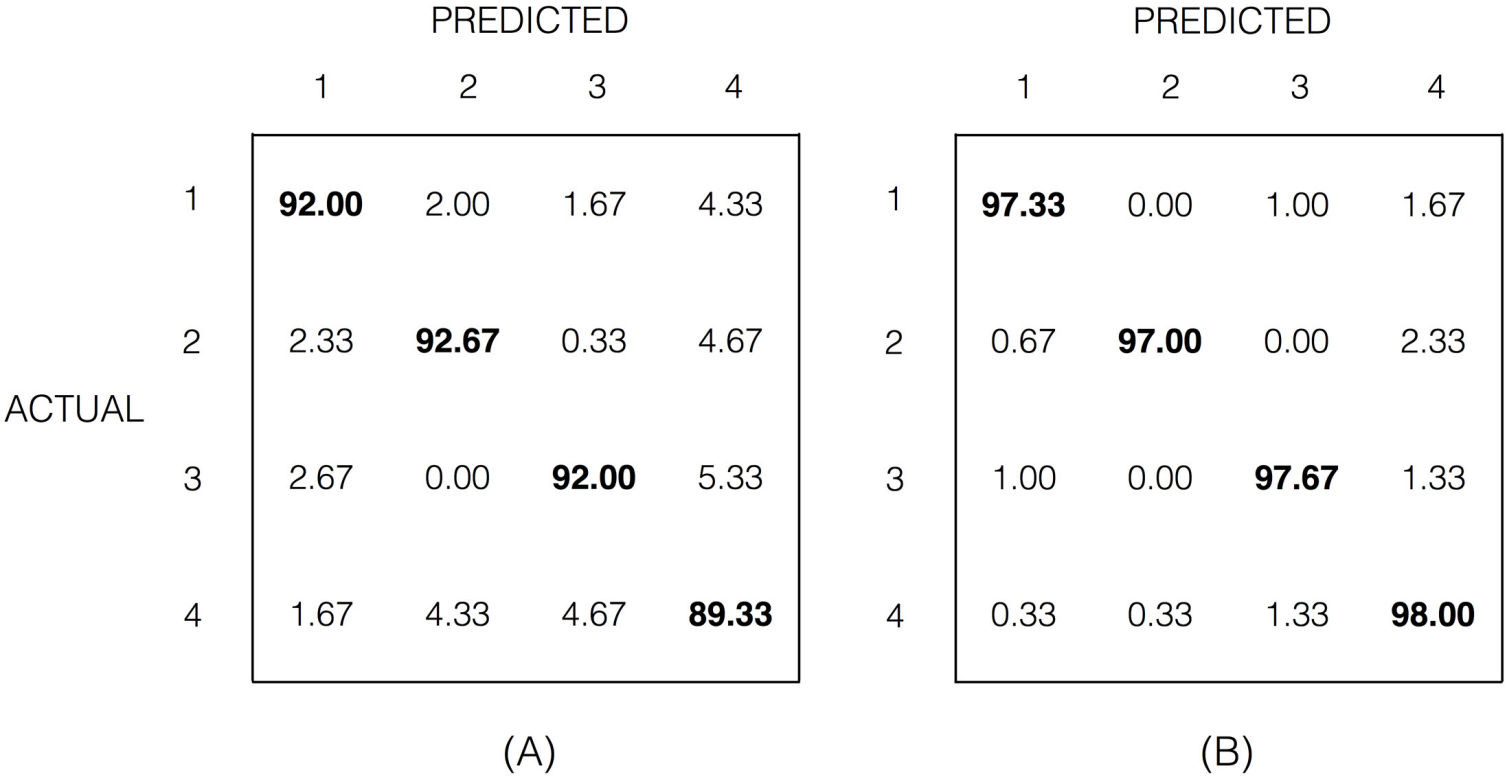
Confusion matrices of the stBF from Fig 7. The numbers on the diagonal indicate the predictive accuracy (in %) of the stBF-classifier for each target. The values displayed are the average of the confusion matrices of all subjects. (A) For an epoch length of 0.5 seconds. (B) For an epoch length of 1 second. Targets 1 and 4 flicker at 12 Hz but with opposite phase, targets 2 and 3 flicker at 15 Hz but with opposite phase.

Finally, Fig 8 shows two confusion matrices of the stBF classifier of Fig 7 for epoch lengths of 0.5 and 1 second. We observe that for target 4 there are more misclassifications (last column in each matrix) than for the other targets and that there are less misclassifications between targets of opposite phase than between targets of different frequencies (*e.g.*, in Fig 8(A), compare predicted target 1 vs. actual target 4 (1.67%) and vs. actual target 2 (2.33%)).

## Discussion

### Effect of combining frequency and phase encoding on SSVEP

Generally, the SSVEP signal has an oscillatory waveform with the same temporal frequency and phase as the flickering stimulus and its harmonics [41] and is considered to be evoked by the electrophysiological activity of large synchronized neuronal populations in early visual cortex [42]. The feasibility of identifying targets from spatially disparate (in visual angle or screen coordinates) flickering stimuli is supported by several lines of evidence. Firstly, as the gaze is directed, the attended stimulus is present in the fovea whereas the unattended targets are in the periphery of the subject’s visual field. As early visual areas are retinotopically organized, disparate visual stimuli activate disparate anatomical locations and vice-versa, as clearly evidenced by several neuroimaging studies [43–45]. When using neuroimaging techniques or EEG in combination with inverse mapping techniques, it was shown that flickering stimuli at different locations in the visual field activate different SSVEP generators in the early visual system [46].

**Fig 9:**
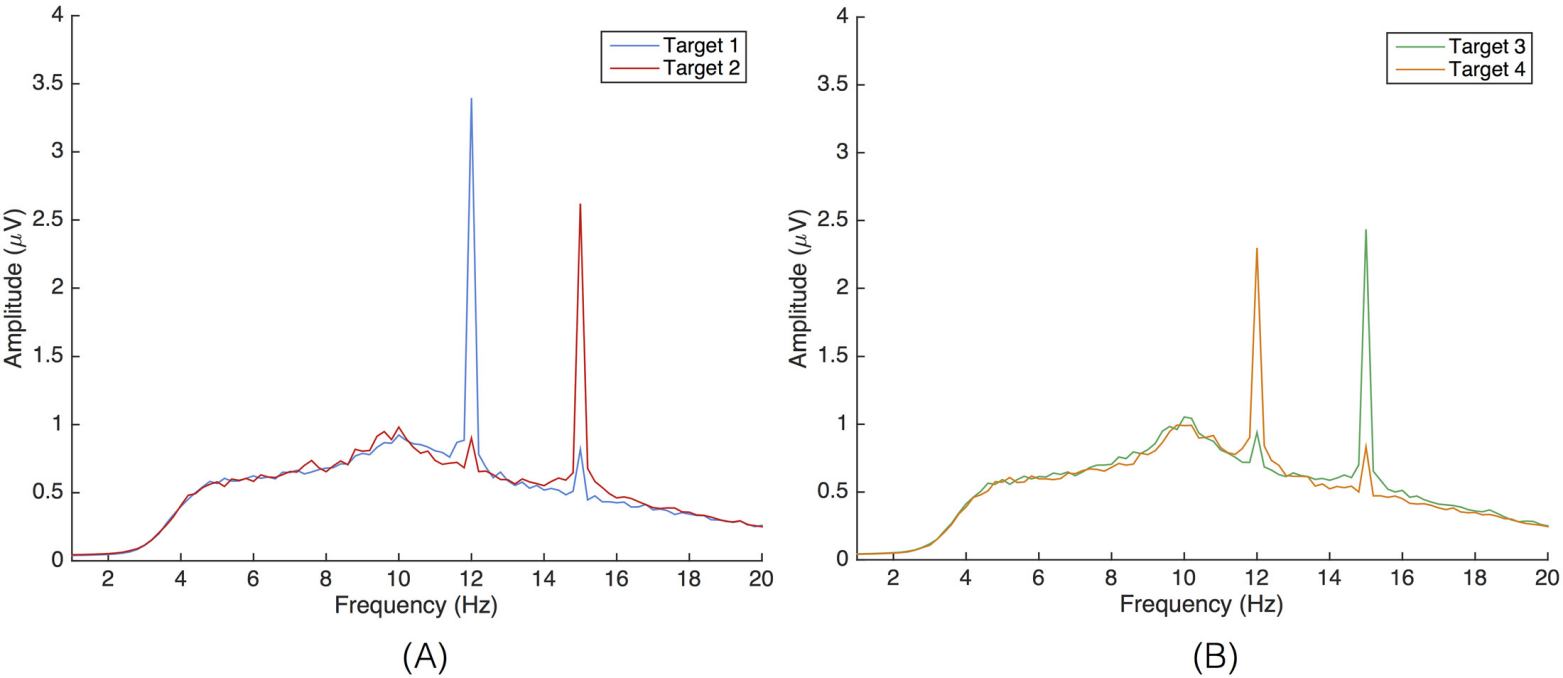
Co-amplification effect. EEG frequency spectrum, averaged over all subjects, revealing co-amplification effect: the peripheral target flickering at a different frequency (∼4.4° separating angle) adds a small amplitude to the SSVEP signal of the attended target. (A) For targets 1 and 2, flickering at 12 and 15 Hz, respectively. (B) For targets 3 and 4, flickering at 15 and 12 Hz, respectively.

A second aspect is the cortical magnification effect: a disproportionately large amount of visual cortex is allotted to processing foveal input compared to more peripheral input [47, 48]. As a result, the electrophysiological response evoked by a stimulus increases when becoming closer to the center of the visual field [48]. This implies that the amplitude of a foveated flickering stimulus is not going to be influenced by an another flickering stimulus when the visual angle that separates them is large enough [49]. When flickering targets are in close proximity, modulatory effects in the recorded EEG signal can be expected, in addition to an effect of attention. This has been studied by Fuchs and co-workers [50]. When the subject is focusing and attending one flickering target, and ignoring the other one, and when the visual angle that separates these targets is smaller than 4°, then the SSVEP amplitude of the attended target is unaltered with a small SSVEP amplitude of the unattended target added to it (co-amplification). Hence, when the flickering frequencies of those two targets are identical, but their phases in opposition (180° phase difference), and their separating angles much smaller than 4°, then the SSVEP amplitude of the attended target will be demoted or even largely cancelled. In our case, te SSVEP responses to attended targets are not canceled by targets with identical frequencies but opposite phases (target 1 vs. 4 and target 2 vs. 3) because their separating angles are much larger (∼6.2°). On the other hand, as the separating angles between targets flickering with different frequencies is much smaller (∼4.4°), we observe a co-amplification effect in the EEG responses to the attended targets (Fig 9). This is also reflected in the confusion matrices (Fig 8) by a much larger degree of misclassification between targets with different flickering frequencies than with opposite phases.

### Activation Patterns

The activation patterns confirm that the SSVEP response is mostly present in the occipital regions, however using only those channels does not yield maximum performance. While surrounding channels have a smaller SSVEP response, they do contain information about noise sources that might contaminate the occipital signals [34]. By taking these channels into account, performance improves. However, increasing the number of channels also increases the dimensionality of the beamformer and the size of the (training) data set needed to accurately estimate the covariance matrix. [35, 36]

The beamformer approach developed in this study constructs an activation pattern for each individual subject, which calls for a training session for each subject. Indeed, the temporal responses to phase-based SSVEP stimulation were observed to vary considerably among subjects (Fig 4), hindering the construction of one activation pattern for the entire population. On the other hand, when individuating the beamformer, it can take into account subject- and session-dependent noise sources, which has a positive effect on performance. In addition, when comparing our beamformers with extended CCA, we observed for the beamformer-based classifiers not only a better performance but also a smaller interquartile range (IQR) over the subject population. This indicates that, although they are developed per subject, the beamformer-based classifiers yield a more stable performance across subjects. While both the beamformer and the extended CCA approach use prior information (targets, subjects), the beamformer takes into account the possible presence of non-target activity and structured noise embedded in the trials by using the covariance matrix, whereas the CCA method only extracts target trials to estimate canonical correlations. We therefore advocate that the beamformer is more efficient in extracting relevant information, leading to a better performance.

### Frequency resolution

**Fig 10:**
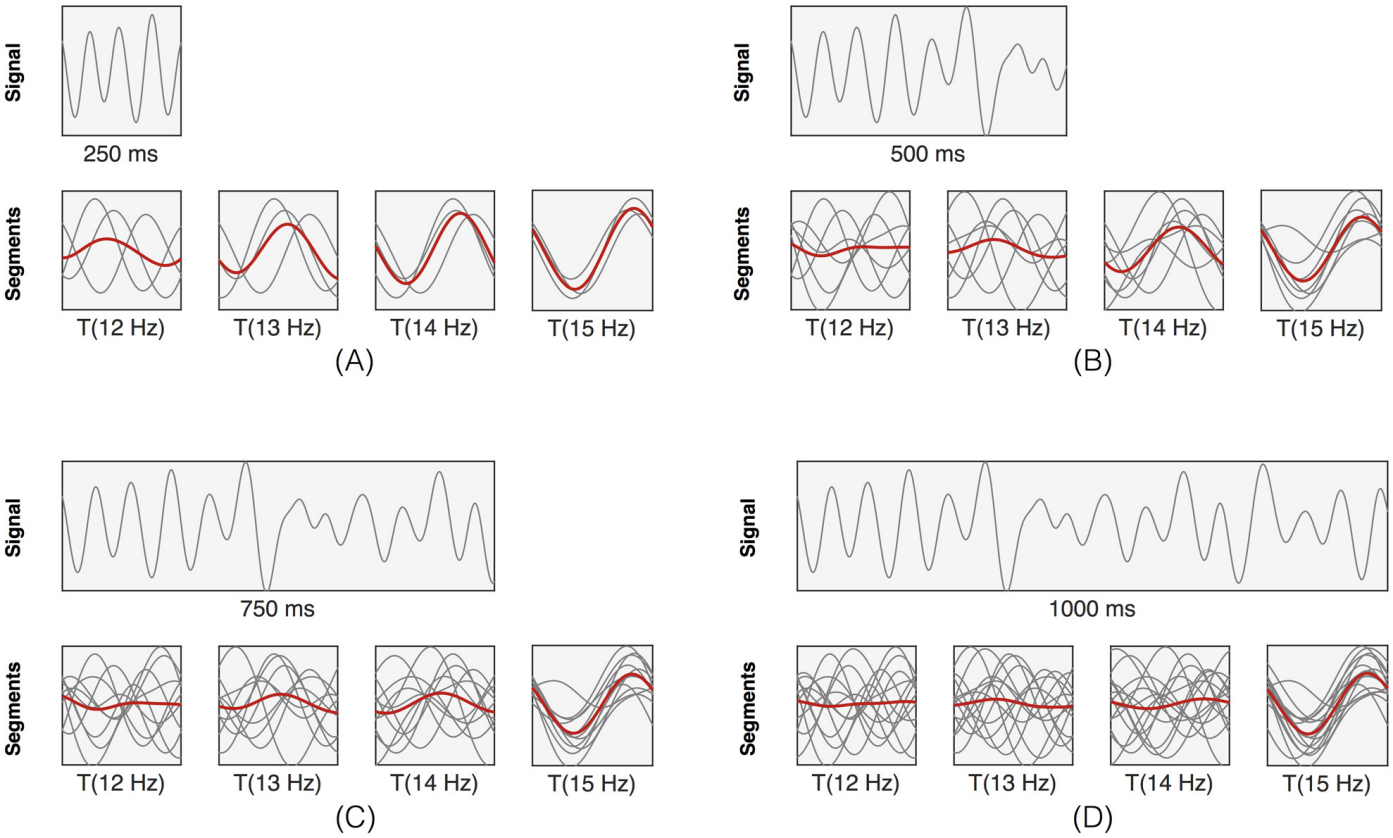
Frequency resolution of the time-domain analysis. A signal from channel Oz in response to a 15 Hz stimulus, and its segments with lengths corresponding to one period (T) of 12, 13, 14 and 15 Hz. Signal length are (A) 250 ms, (B) 500 ms, (C) 750 ms and (D) 1000 ms.

Our time-domain analysis of SSVEP signals is influenced by the frequencies used for stimulating the targets. If the difference in segment lengths between targets increases then less segments will be needed to accurately discriminate between a waveform and a ‘flat’ response. Fig 10 shows the effect of epoch lengths for a 15 Hz SSVEP response at channel *Oz* when assuming targets with frequencies (12, 13, 14, and 15 Hz). It is clear that longer epoch lengths are needed when discriminating targets with similar stimulation frequencies. Indeed, for 250 ms, one can easily exclude the 12 Hz response from the list of possible target responses, but longer segments are needed to discriminate between the 14 and 15 Hz responses.

## Conclusion

In this study, we have introduced beamforming as a new method for simultaneous frequency and phase detection in SSVEP-based BCI application. We have shown that our method outperforms an extension of CCA which has been touted as the best method for targets encoded by individual frequency-phase combinations, even for 1.25s epoch lengths.

## Supporting Information

S1 Table P-values for the performance differences of the chBF-based classifier, using different channel sets. Values are calculated using a two-sided Wilcoxon Rank-Sum Test. Significant values are indicates in bold.
(PDF)

S2 Table P-values for the performance differences of the stBF-based classifier, using different channel sets. Values are calculated using a two-sided Wilcoxon Rank-Sum Test. Significant values are indicates in bold.
(PDF)

S3 Table P-values for the performance differences of the chBF-based classifier, using different downsampling rates. Values are calculated using a two-sided Wilcoxon Rank-Sum Test. Significant values are indicates in bold.
(PDF)

S4 Table P-values for the performance differences of the stBF-based classifier, using different downsampling rates. Values are calculated using a two-sided Wilcoxon Rank-Sum Test. Significant values are indicates in bold.
(PDF)

S5 Table P-values for the performance differences between classifiers. A downsampling rate of 512 Hz and channel set Ch_env_ was used. Values are calculated using a two-sided Wilcoxon Rank-Sum Test. Significant values are indicates in bold.
(PDF)

S1 Fig Accuracies of the four sub-classifiers based on stBF. A downsampling rate of 512 Hz and channel set Ch_env_ was used.
(PDF)

S2 Fig Accuracies of the four sub-classifiers based on chBF. A downsampling rate of 512 Hz and channel set Ch_env_ was used.
(PDF)

## References

[1] Cheng M, Gao X, Gao S, Xu D. Design and implementation of a brain-computer interface with high transfer rates. Biomedical Engineering, IEEE Transactions on. 2002;49(10):1181–1186. 10.1109/TBME.2002.803536 12374343

[2] Kelly SP, Lalor EC, Reilly RB, Foxe JJ. Visual spatial attention tracking using high-density SSVEP data for independent brain-computer communication. Neural Systems and Rehabilitation Engineering, IEEE Transactions on. 2005;13(2):172–178. 10.1109/TNSRE.2005.847369 16003896

[3] Wu CH, Chang HC, Lee PL, Li KS, Sie JJ, Sun CW, et al Frequency recognition in an SSVEP-based brain computer interface using empirical mode decomposition and refined generalized zero-crossing. Journal of Neuroscience Methods. 2011;196(1):170–181. Available from: http://www.sciencedirect.com/science/article/pii/S0165027010007028. 10.1016/j.jneumeth.2010.12.014 21194547

[4] Liu Q, Chen K, Ai Q, Xie SQ. Review: recent development of signal processing algorithms for SSVEP-based brain computer interfaces. J Med Biol Eng. 2013;.

[5] Wu Z. SSVEP Extraction Based on the Similarity of Background EEG. PloS one. 2014;9(4):e93884 10.1371/journal.pone.0093884 24709951 PMC3977932

[6] Friman O, Volosyak I, Gräser A. Multiple channel detection of steady-state visual evoked potentials for brain-computer interfaces. Biomedical Engineering, IEEE Transactions on. 2007;54(4):742–750. 10.1109/TBME.2006.889160 17405382

[7] Luo A, Sullivan TJ. A user-friendly SSVEP-based brain–computer interface using a time-domain classifier. Journal of neural engineering. 2010;7(2):026010 10.1088/1741-2560/7/2/026010 20332551

[8] Manyakov NV, Chumerin N, Combaz A, Robben A, Van Hulle MM. Decoding SSVEP Responses using Time Domain Classification. In: IJCCI (ICFC-ICNC); 2010. p. 376–380.

[9] Lin Z, Zhang C, Wu W, Gao X. Frequency recognition based on canonical correlation analysis for SSVEP-based BCIs. Biomedical Engineering, IEEE Transactions on. 2006;53(12):2610–2614. 10.1109/TBME.2006.886577 17152442

[10] Bin G, Gao X, Yan Z, Hong B, Gao S. An online multi-channel SSVEP-based brain–computer interface using a canonical correlation analysis method. Journal of neural engineering. 2009;6(4):046002 10.1088/1741-2560/6/4/046002 19494422

[11] Xie J, Xu G, Wang J, Zhang F, Zhang Y. Steady-State Motion Visual Evoked Potentials Produced by Oscillating Newton’s Rings: Implications for Brain-Computer Interfaces. PLoS ONE. 2012 06;7(6):e39707 Available from: http://dx.doi.org/10.1371%2Fjournal.pone.0039707. 10.1371/journal.pone.0039707 22724028 PMC3378577

[12] Yin E, Zhou Z, Jiang J, Yu Y, Hu D. A Dynamically Optimized SSVEP Brain-Computer Interface (BCI) Speller. Biomedical Engineering, IEEE Transactions on. 2015 6;62(6):1447–1456. 10.1109/TBME.2014.2320948 24801483

[13] Zhang Y, Zhou G, Jin J, Wang X, Cichocki A. Frequency recognition in SSVEP-based BCI using multiset canonical correlation analysis. International journal of neural systems. 2014;24(04):1450013 10.1142/S0129065714500130 24694168

[14] Chen X, Wang Y, Gao S, Jung TP, Gao X. Filter bank canonical correlation analysis for implementing a high-speed SSVEP-based brain–computer interface. Journal of neural engineering. 2015;12(4):046008 10.1088/1741-2560/12/4/046008 26035476

[15] Manyakov NV, Chumerin N, Robben A, Combaz A, van Vliet M, Van Hulle MM. Sampled sinusoidal stimulation profile and multichannel fuzzy logic classification for monitor-based phase-coded SSVEP brain–computer interfacing. Journal of neural engineering. 2013;10(3):036011 10.1088/1741-2560/10/3/036011 23594762

[16] Lee PL, Sie JJ, Liu YJ, Wu CH, Lee MH, Shu CH, et al An SSVEP-actuated brain computer interface using phase-tagged flickering sequences: a cursor system. Annals of Biomedical Engineering. 2010;38(7):2383–2397. 10.1007/s10439-010-9964-y 20177780

[17] Lopez-Gordo M, Prieto A, Pelayo F, Morillas C. Use of phase in brain–computer interfaces based on steady-state visual evoked potentials. Neural processing letters. 2010;32(1):1–9. 10.1007/s11063-010-9139-8

[18] Zhu D, Garcia-Molina G, Mihajlović V, Aarts RM. Online BCI implementation of high-frequency phase modulated visual stimuli In: Universal Access in Human-Computer Interaction. Users Diversity. Springer; 2011 p. 645–654.

[19] Falzon O, Camilleri K, Muscat J. Complex-valued spatial filters for SSVEP-based BCIs with phase coding. Biomedical Engineering, IEEE Transactions on. 2012;59(9):2486–2495. 10.1109/TBME.2012.2205246 22736630

[20] Manyakov N, Chumerin N, Combaz A, Robben A, van Vliet M, Van Hulle M. Decoding Phase-Based Information from Steady-State Visual Evoked Potentials with Use of Complex-Valued Neural Network. In: Yin H, Wang W, Rayward-Smith V, editors. Intelligent Data Engineering and Automated Learning—IDEAL 2011. vol. 6936 of Lecture Notes in Computer Science. Springer Berlin Heidelberg; 2011 p. 135–143. Available from: 10.1007/978-3-642-23878-9_17.

[21] Manyakov NV, Chumerin N, Van Hulle MM. Multichannel decoding for phase-coded SSVEP brain–computer interface. International journal of neural systems. 2012;22(05):1250022 10.1142/S0129065712500220 22963395

[22] Jia C, Gao X, Hong B, Gao S. Frequency and phase mixed coding in SSVEP-based brain–computer interface. Biomedical Engineering, IEEE Transactions on. 2011;58(1):200–206. 10.1109/TBME.2010.2068571 20729160

[23] Chen X, Wang Y, Nakanishi M, Jung TP, Gao X. Hybrid frequency and phase coding for a high-speed SSVEP-based BCI speller. In: Engineering in Medicine and Biology Society (EMBC), 2014 36th Annual International Conference of the IEEE. IEEE; 2014. p. 3993–3996. 10.1109/EMBC.2014.6944499 25570867

[24] Nakanishi M, Wang Y, Wang YT, Mitsukura Y, Jung TP. A high-speed brain speller using steady-state visual evoked potentials. International journal of neural systems. 2014;24(06):1450019 10.1142/S0129065714500191 25081427

[25] Van Veen BD, Buckley KM. Beamforming: A versatile approach to spatial filtering. IEEE assp magazine. 1988;5(2):4–24. 10.1109/53.665

[26] Van Veen BD, Van Drongelen W, Yuchtman M, Suzuki A. Localization of brain electrical activity via linearly constrained minimum variance spatial filtering. Biomedical Engineering, IEEE Transactions on. 1997;44(9):867–880. 10.1109/10.623056 9282479

[27] van Vliet M, Chumerin N, De Deyne S, Wiersema JR, Fias W, Storms G, et al Single-trial ERP component analysis using a spatio-temporal LCMV beamformer. Biomedical Engineering, IEEE Transactions on. 2015;PP(99):1–1. 10.1109/TBME.2015.2468588 26285053

[28] Wittevrongel B, Van Hulle MM. Faster P300 Classifier Training Using Spatiotemporal Beamforming. International Journal of Neural Systems. 2016;26(3):1650014 10.1142/S0129065716500143 26971787

[29] Grosse-Wentrup M, Liefhold C, Gramann K, Buss M. Beamforming in noninvasive brain–computer interfaces. Biomedical Engineering, IEEE Transactions on. 2009;56(4):1209–1219. 10.1109/TBME.2008.2009768 19423426

[30] Croft R, Barry R. Removal of ocular artifact from the EEG: a review. Neurophysiologie Clinique/Clinical Neurophysiology. 2000;30(1):5–19. 10.1016/S0987-7053(00)00055-1 10740792

[31] Tobimatsu S, Tomoda H, Kato M. Normal variability of the amplitude and phase of steady-state VEPs. Electroencephalography and Clinical Neurophysiology/Evoked Potentials Section. 1996;100(3):171–176. 10.1016/0168-5597(95)00279-0 8681857

[32] Di Russo F, Spinelli D. Electrophysiological evidence for an early attentional mechanism in visual processing in humans. Vision research. 1999;39(18):2975–2985. 10.1016/S0042-6989(99)00031-0 10664797

[33] Di Russo F, Teder-Sälejärvi WA, Hillyard SA. Steady-state VEP and attentional visual processing The cognitive electrophysiology of mind and brain (Zani A, Proverbio AM, eds). 2002;p. 259–274.

[34] Haufe S, Meinecke F, Görgen K, Dähne S, Haynes JD, Blankertz B, et al On the interpretation of weight vectors of linear models in multivariate neuroimaging. Neuroimage. 2014;87:96–110. 10.1016/j.neuroimage.2013.10.067 24239590

[35] Pruzek RM. High Dimensional Covariance Estimation: Avoiding the ‘Curse of Dimensionality’ In: Proceedings of the First US/Japan Conference on the Frontiers of Statistical Modeling: An Informational Approach. Springer; 1994 p. 233–253.

[36] Schoukens J, Pintelon R. Identification of linear systems: a practical guideline to accurate modeling. Elsevier; 2014.

[37] Combaz A, Chatelle C, Robben A, Vanhoof G, Goeleven A, Thijs V, et al A comparison of two spelling brain-computer interfaces based on visual P3 and SSVEP in Locked-In Syndrome. PloS one. 2013;8(9):e73691 10.1371/journal.pone.0073691 24086289 PMC3783473

[38] Brunner C, Allison B, Altstätter C, Neuper C. A comparison of three brain-computer interfaces based on event-related desynchronization, steady state visual evoked potentials, or a hybrid approach using both signals. Journal of neural engineering. 2011;8(2):025010 10.1088/1741-2560/8/2/025010 21436538

[39] Kübler A, Neumann N, Wilhelm B, Hinterberger T, Birbaumer N. Predictability of brain-computer communication. Journal of Psychophysiology. 2004;18(2/3):121–129. 10.1027/0269-8803.18.23.121

[40] Kübler A, Birbaumer N. Brain-computer interfaces and communication in paralysis: Extinction of goal directed thinking in completely paralysed patients? Clinical neurophysiology. 2008;119(11):2658–2666. 10.1016/j.clinph.2008.06.019 18824406 PMC2644824

[41] Regan D. Human brain electrophysiology: evoked potentials and evoked magnetic fields in science and medicine. Elsevier; 1989.

[42] Rager G, Singer W. The response of cat visual cortex to flicker stimuli of variable frequency. European Journal of Neuroscience. 1998;10(5):1856–1877. 10.1046/j.1460-9568.1998.00197.x 9751156

[43] Bridge H. Mapping the visual brain: how and why. Eye. 2011;25(3):291–296. 10.1038/eye.2010.166 21102491 PMC3178304

[44] Vanegas MI, Blangero A, Kelly SP. Exploiting individual primary visual cortex geometry to boost steady state visual evoked potentials. Journal of neural engineering. 2013;10(3):036003 10.1088/1741-2560/10/3/036003 23548662 PMC3660541

[45] Raz N, Levin N. Cortical and white matter mapping in the visual system-more than meets the eye: on the importance of functional imaging to understand visual system pathologies. Neurovision: Neural bases of binocular vision and coordination and their implications in visual training programs. 2015;p. 90. 10.3389/fnint.2014.00068 PMC4145715 25221482

[46] Di Russo F, Pitzalis S, Aprile T, Spitoni G, Patria F, Stella A, et al Spatiotemporal analysis of the cortical sources of the steady-state visual evoked potential. Human brain mapping. 2007;28(4):323–334. 10.1002/hbm.20276 16779799 PMC6871301

[47] Cowey A, Rolls E. Human cortical magnification factor and its relation to visual acuity. Experimental Brain Research. 1974;21(5):447–454. 10.1007/BF00237163 4442497

[48] Sutter EE. The brain response interface: communication through visually-induced electrical brain responses. Journal of Microcomputer Applications. 1992;15(1):31–45. 10.1016/0745-7138(92)90045-7

[49] Zhao J, Zhang Z, Zhang C, Tang Y, Liu Z. Neural suppression of distractors surrounding the spotlight: Evidence from steady-state visual evoked potentials. Chinese Science Bulletin. 2012;57(14):1680–1684. 10.1007/s11434-012-5078-2

[50] Fuchs S, Andersen SK, Gruber T, Müller MM. Attentional bias of competitive interactions in neuronal networks of early visual processing in the human brain. NeuroImage. 2008;41(3):1086–1101. 10.1016/j.neuroimage.2008.02.040 18424083

