# Supplementary material for: Frequency- and Phase Encoded SSVEP Using Spatiotemporal Beamforming
Source: PLoS One. 2016 Aug 3;11(8):e0159988. doi: 10.1371/journal.pone.0159988 (PMC4972379; doi:10.1371/journal.pone.0159988)
Supplement: S1 Table — Values are calculated using a two-sided Wilcoxon Rank-Sum Test. Significant values are indicates in bold. (PDF) [file pone.0159988.s001.pdf]

S1 Table. P-values for the performance differences of the chBF-based classifier, using different channel sets. Values are calculated using a two-sided Wilcoxon Rank-Sum Test. Significant values are indicates in bold.

Channel Sets	Epoch Length (s)											
	0.25	*0.50*	*0.75*	*1.0*	*1.25*	*1.50*	*1.75*	*2.00*	*2.25*	*2.50*	*2.75*	*3.0*
Ch_{full} - Ch_{env}	0.064	0.086	0.174	0.814	0.727	0.819	0.837	0.768	0.368	0.582	0.914	0.852
Ch_{full} - Ch_{occ}	0.317	0.783	0.316	0.349	0.160	0.094	0.086	0.130	0.213	0.234	0.360	0.257
Ch_{env} - Ch_{occ}	0.597	0.079	0.063	0.265	0.281	0.112	0.083	0.066	0.647	0.493	0.312	0.196
