# Supplementary material for: Frequency- and Phase Encoded SSVEP Using Spatiotemporal Beamforming
Source: PLoS One. 2016 Aug 3;11(8):e0159988. doi: 10.1371/journal.pone.0159988 (PMC4972379; doi:10.1371/journal.pone.0159988)
Supplement: S2 Table — Values are calculated using a two-sided Wilcoxon Rank-Sum Test. Significant values are indicates in bold. (PDF) [file pone.0159988.s002.pdf]

S2 Table. P-values for the performance differences of the stBF-based classifier, using different channel sets. Values are calculated using a two-sided Wilcoxon Rank-Sum Test. Significant values are indicates in bold.

Channel Sets	Epoch Length (s)											
	0.25	*0.50*	*0.75*	*1.0*	*1.25*	*1.50*	*1.75*	*2.00*	*2.25*	*2.50*	*2.75*	*3.0*
Ch_{full} - Ch_{env}	**0.004**	**<0.001**	**<0.001**	**<0.001**	**<0.001**	**<0.001**	**<0.001**	**<0.001**	**<0.001**	0.051	0.051	0.115
Ch_{full} - Ch_{occ}	**<0.001**	**<0.001**	**<0.001**	**<0.001**	**<0.001**	**<0.001**	**<0.001**	**<0.001**	**0.011**	0.139	0.302	0.510
Ch_{env} - Ch_{occ}	**<0.001**	0.662	0.973	0.280	0.314	0.079	0.507	0.280	0.266	0.606	0.354	0.345
