# Supplementary material for: Frequency- and Phase Encoded SSVEP Using Spatiotemporal Beamforming
Source: PLoS One. 2016 Aug 3;11(8):e0159988. doi: 10.1371/journal.pone.0159988 (PMC4972379; doi:10.1371/journal.pone.0159988)
Supplement: S3 Table — Values are calculated using a two-sided Wilcoxon Rank-Sum Test. Significant values are indicates in bold. (PDF) [file pone.0159988.s003.pdf]

S3 Table. P-values for the performance differences of the chBF-based classifier, using different downsampling rates. Values are calculated using a two-sided Wilcoxon Rank-Sum Test. Significant values are indicates in bold.

Downsampling (Hz)	Epoch Length (s)											
	0.25	*0.50*	*0.75*	*1.0*	*1.25*	*1.50*	*1.75*	*2.00*	*2.25*	*2.50*	*2.75*	*3.0*
512 - 256	0.682	0.825	0.584	0.855	0.720	0.485	0.511	0.364	0.991	0.626	0.735	0.865
512 - 128	0.222	0.357	0.243	0.479	0.771	0.312	0.231	0.064	0.305	0.366	0.591	0.458
256 - 128	0.337	0.569	0.479	0.522	0.941	0.657	0.555	0.332	0.294	0.667	0.895	0.604
