# Supplementary material for: Frequency- and Phase Encoded SSVEP Using Spatiotemporal Beamforming
Source: PLoS One. 2016 Aug 3;11(8):e0159988. doi: 10.1371/journal.pone.0159988 (PMC4972379; doi:10.1371/journal.pone.0159988)
Supplement: S4 Table — Values are calculated using a two-sided Wilcoxon Rank-Sum Test. Significant values are indicates in bold. (PDF) [file pone.0159988.s004.pdf]

S4 Table. P-values for the performance differences of the stBF-based classifier, using different downsampling rates Values are calculated using a two-sided Wilcoxon Rank-Sum Test. Significant values are indicates in bold.

Downsampling (Hz)	Epoch Length (s)											
	0.25	*0.50*	*0.75*	*1.0*	*1.25*	*1.50*	*1.75*	*2.00*	*2.25*	*2.50*	*2.75*	*3.0*
512 - 256	0.175	0.238	0.502	0.462	0.706	0.678	0.871	0.918	0.797	1.0	0.965	0.763
512 - 128	**0.003**	0.752	0.602	0.859	0.898	0.854	0.973	0.765	0.600	0.876	1.0	0.899
256 - 128	<**0.001**	0.391	0.940	0.604	0.808	0.806	0.790	0.849	0.769	0.843	1.0	0.826
