# Supplementary material for: Frequency- and Phase Encoded SSVEP Using Spatiotemporal Beamforming
Source: PLoS One. 2016 Aug 3;11(8):e0159988. doi: 10.1371/journal.pone.0159988 (PMC4972379; doi:10.1371/journal.pone.0159988)
Supplement: S5 Table — A downsampling rate of 512 Hz and channel set Chenv was used. Values are calculated using a two-sided Wilcoxon Rank-Sum Test. Significant values are indicates in bold. (PDF) [file pone.0159988.s005.pdf]

S5 Table. P-values for the performance differences between classifiers. A downsampling rate of 512 Hz and channel set Ch_{env} was used. Values are calculated using a two-sided Wilcoxon Rank-Sum Test. Significant values are indicates in bold.

Classifiers	Epoch Length (s)											
	0.25	*0.50*	*0.75*	*1.0*	*1.25*	*1.50*	*1.75*	*2.00*	*2.25*	*2.50*	*2.75*	*3.0*
chBF - stBF	<**0.001**	0.653	0.930	**0.023**	**0.042**	0.181	0.351	0.740	0.240	0.244	0.438	0.521
chBF - CCA	<**0.001**	**0.002**	0.061	0.496	0.554	0.400	0.140	**0.048**	0.212	0.650	0.606	0.258
stBF - CCA	0.097	<**0.001**	**0.023**	**0.003**	**0.019**	0.056	**0.022**	**0.018**	**0.022**	0.170	0.232	0.081
