# Supplementary figures and images for: Frequency- and Phase Encoded SSVEP Using Spatiotemporal Beamforming
Source: PLoS One. 2016 Aug 3;11(8):e0159988. doi: 10.1371/journal.pone.0159988 (PMC4972379; doi:10.1371/journal.pone.0159988)

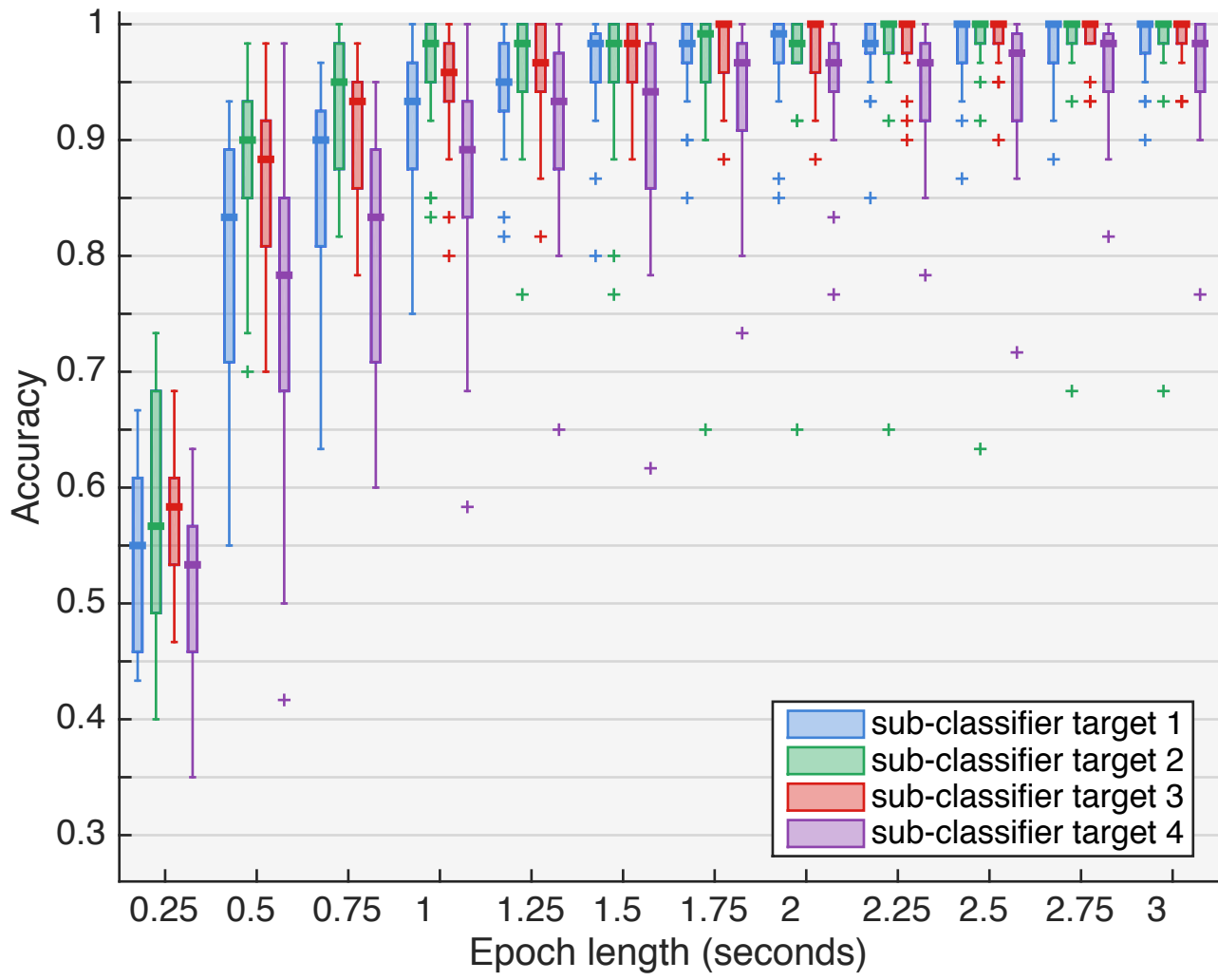

Supplement: S1 Fig — A downsampling rate of 512 Hz and channel set Chenv was used. (PDF) [file pone.0159988.s006.pdf]

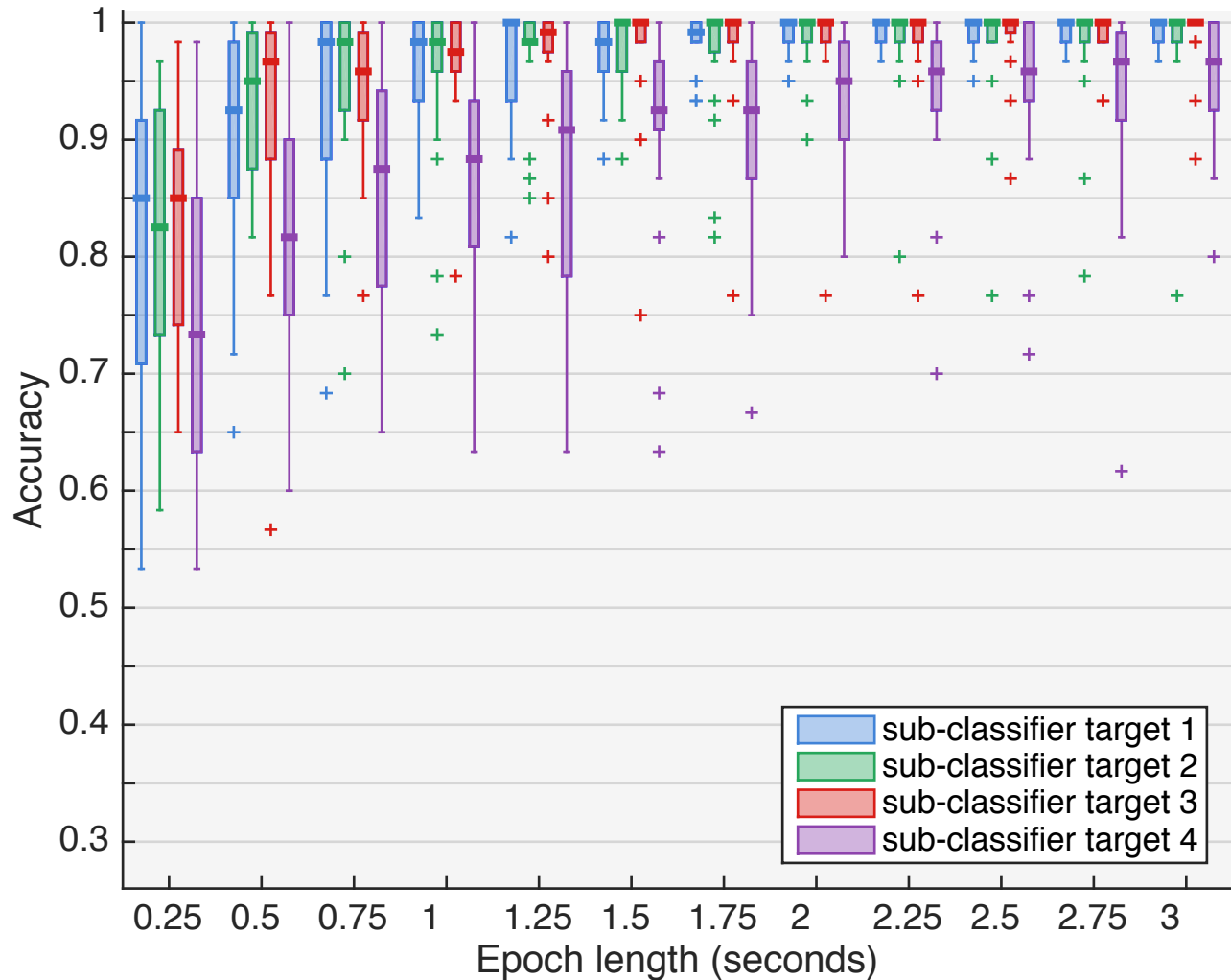

Supplement: S2 Fig — A downsampling rate of 512 Hz and channel set Chenv was used. (PDF) [file pone.0159988.s007.pdf]
